# Tourism-supported working lands sustain a growing jaguar population in the Colombian Llanos

**DOI:** 10.1038/s41598-023-36935-2

**Published:** 2023-06-27

**Authors:** Matthew Hyde, Esteban Payán, Jorge Barragan, Diana Stasiukynas, Samantha Rincón, William L. Kendall, Jerónimo Rodríguez, Kevin R. Crooks, Stewart W. Breck, Valeria Boron

**Affiliations:** 1 Graduate Degree Program in Ecology, Center for Human-Carnivore Coexistence, Colorado State University, 112 Wagar Building, Fort Collins, CO 80523 USA; 2 Panthera, 8 W 40th St, 18th Floor, New York, NY 10018 USA; 3 Wildlife Conservation Society, NY Bronx, USA; 4 Reserva Natural de la Sociedad Civil Hato La Aurora, Fundación Jaguar Colombia, Hato Corozal, Casanare, Colombia; 5 U.S. Geological Survey, Colorado Cooperative Fish and Wildlife Research Unit, Fort Collins, CO 80523 USA; 6 Department of Fish, Wildlife, and Conservation Biology, Colorado State University, Fort Collins, CO USA; 7 National Wildlife Research Center, United States Department of Agriculture (USDA)-Wildlife Services, Fort Collins, CO USA; 8 World Wide Fund For Nature (WWF) UK, The Living Planet Centre, Rufford House Brewery Road, Woking, GU21 4LL Surrey UK

**Keywords:** Biodiversity, Conservation biology, Population dynamics, Tropical ecology

## Abstract

Understanding large carnivore demography on human-dominated lands is a priority to inform conservation strategies, yet few studies examine long-term trends. Jaguars (*Panthera onca*) are one such species whose population trends and survival rates remain unknown across working lands. We integrated nine years of camera trap data and tourist photos to estimate jaguar density, survival, abundance, and probability of tourist sightings on a working ranch and tourism destination in Colombia. We found that abundance increased from five individuals in 2014 to 28 in 2022, and density increased from 1.88 ± 0.87 per 100 km^2^ in 2014 to 3.80 ± 1.08 jaguars per 100 km^2^ in 2022. The probability of a tourist viewing a jaguar increased from 0% in 2014 to 40% in 2020 before the Covid-19 pandemic. Our results are the first robust estimates of jaguar survival and abundance on working lands. Our findings highlight the importance of productive lands for jaguar conservation and suggest that a tourism destination and working ranch can host an abundant population of jaguars when accompanied by conservation agreements and conflict interventions. Our analytical model that combines conventional data collection with tourist sightings can be applied to other species that are observed during tourism activities.

Entender los patrones demográficos de los grandes carnívoros al interior de paisajes antrópicos es fundamental para el diseño de estrategias de conservación efectivas. En el Neotrópico, el jaguar (*Panthera onca*) es una de estas especies cuyas tendencias poblacionales y tasas de supervivencia en paisajes productivos son desconocidas. Para entender mejor estas dinámicas, integramos nueve años de fototrampeo junto a fotos de turistas para estimar la densidad, supervivencia, abundancia y probabilidad de avistamiento de esta especie en una finca ganadera y destino turístico en Colombia. Entre 2014 y 2022 encontramos que la abundancia incrementó de cinco a 28 individuos y la densidad de 1.88 ± 0.87 jaguares/ 100 km^2^ a 3.80 ± 1.08 jaguares/ 100 km^2^. La probabilidad de avistamiento por turistas aumentó de 0% en 2014 a 40% en 2020 antes de la pandemia del Covid-19. Nuestros resultados presentan las primeras estimaciones robustas de abundancia y supervivencia de este felino en paisajes antrópicos dónde el manejo de sistemas productivos combinados con turismo e intervenciones para la mitigación del conflicto puede albergar poblaciones abundantes de jaguares, demostrando su importancia para la conservación de esta especie. Nuestro modelo, al combinar datos convencionales con avistamientos, podría ser aplicado a otras especies observadas durante actividades turísticas.

## Introduction

Large carnivores can play crucial regulatory roles in the ecosystems they inhabit, yet populations of many species continue to decline despite conservation efforts^1^. A key strategy for conserving large carnivores is reducing pressures in human-dominated landscapes, given that most suitable habitat occurs outside of protected areas^2,3^. Working lands can provide vital, permeable habitat for carnivores^4^. However, interactions between large carnivores and humans on private lands may be negative, impacting both carnivores and people^5^. Conservation scientists and practitioners are interested in developing strategies to promote coexistence with large carnivores^6^, where carnivore populations are sustained and human livelihoods thrive with minimal conflict^7^.

Whereas local support for carnivore presence on working lands tends to be low due to livestock depredation and safety concerns, value for carnivores at the global scale, especially felids, tends to be high^8^. Successful coexistence with large carnivores requires converting global existence value into tangible financial benefits for local communities^9^. Conservationists have explored various funding mechanisms to create value from the presence of large carnivores for rural communities^9,10^. One such opportunity to promote coexistence locally and generate income for those living with carnivores is wildlife tourism, where tourists pay to observe fauna in their natural habitats.

Because attitudes towards large carnivores are a strong predictor of human behavior towards them^11,12^, proponents hope that tourism income can sway local communities towards positive perceptions of carnivores while benefiting wildlife populations^13^. Income from wildlife tourism can offset the costs of living with large carnivores, such as livestock depredation^14^. Furthermore, wildlife tourism can contribute to conservation through funding anti-poaching patrols, livestock compensation programs, and species recovery and restoration, as well as providing opportunities for scientific research and wildlife monitoring^13,15^. However, wildlife tourism initiatives can have both positive and negative outcomes for wildlife populations^16–18^. The success of coexistence strategies like tourism requires understanding and quantifying such impacts^19^, and these strategies promote coexistence more effectively when accompanied by efforts to reduce livestock losses and conservation agreements^20^. Moreover, the presence of tourists with high-quality photography capabilities can have important, albeit underutilized, contributions to wildlife monitoring^21^.

Developing effective coexistence strategies for jaguars (*Panthera onca*) is urgently needed. Jaguars are among the most emblematic large carnivores^22^. Over half (55%) of remaining populations exist outside of protected areas^23^, hence research and conservation strategies on working lands are of critical importance^24^. Jaguars have lost nearly half their range in the last 50 years^25^. Major threats to their persistence are habitat loss and fragmentation^26^, direct killings to prevent or retaliate for livestock losses, and prey depletion^25^.

Understanding jaguar survival rates is critical to their conservation, but acquiring accurate population metrics can be difficult and costly^27^. Jaguars are naturally elusive, and densities are low^23^. Longitudinal studies of jaguars are therefore challenging because of the financial and logistical difficulty of long-term monitoring. Across the jaguar’s extensive range, only four long-term jaguar population studies have been published^27–30^. In addition, no published long-term study has addressed survival and abundance on working lands, where densities tend to be lower^23^ and survival is likely more challenging.

Hato La Aurora, a working cattle ranch and private reserve, is the only place in Colombia where jaguar tourism is practiced in the species’ natural habitat. Hato La Aurora was formally established as a reserve in 2008, and hunting was prohibited on the ranch since 1979. Because of the killing of jaguars for their pelts (known locally as *tigrilladas*) in the mid-twentieth century^31^, populations were decimated, and no jaguars were observed in Hato La Aurora until 2002. In 2013, Hato La Aurora began working with Panthera Colombia, a felid conservation organization, to monitor the jaguar population and reduce human-jaguar conflict in and around the ranch. Panthera Colombia implemented conservation agreements and electric fences around calving pastures to reduce livestock depredations from jaguars with 19 ranches neighboring Hato La Aurora, totaling 8558 hectares contiguous to Hato La Aurora and 19,326 hectares nearby (Panthera Colombia, unpublished data).

Here, we present the first longitudinal study and demographic estimates for jaguars on working lands and in Colombia. We integrated nine years of camera trap data and tourist photos of jaguars collected between 2014 and 2022 in a Barker Robust Design Model (Barker/RD)^32^. Our approach estimated survival rates, probability of observation by tourists, and abundance of jaguars, and we derived finite rate of change in abundance and recruitment^33^. We complemented the robust design analysis by comparing our results with spatially explicit density estimates in 2014 and 2022 from the study area. We hypothesized that jaguar abundance, survival, density, and observation by tourists increased over time as a result of recently implemented conservation efforts. We discuss how our findings contribute to human-jaguar coexistence strategies, and how our modeling approach can contribute to demographic studies of species photographed by tourists.

## Results

**Figure 1:**
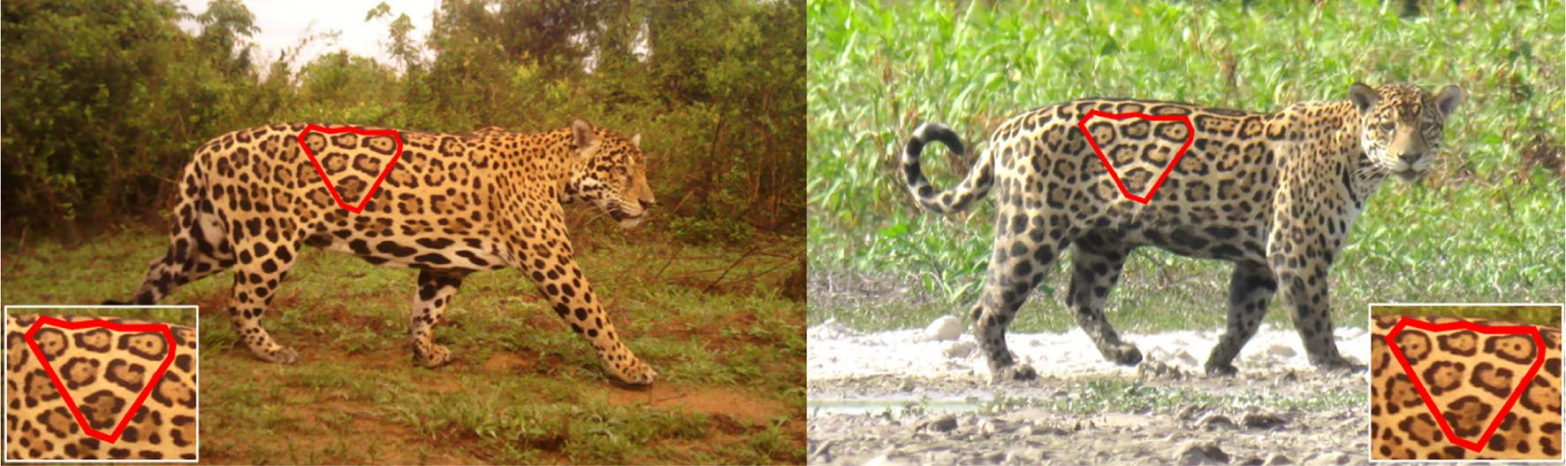
Example of a camera trap detection (left) and a tourist sighting (right) of the same individual. Photos: Fundación Jaguar & Panthera Colombia (left), S. Rincón (right).

We recorded 50 individual jaguars from 659 identifiable camera trap records and 79 tourist sightings between 2014 and 2022 (Fig. 1). We detected 19 females and 31 males, although five individuals (four of which were females) were removed from the analysis because they were not detected within closed-capture time periods.

We ran 26 models in the Barker/RD to explore our hypotheses (Table A1), three of which summed to 0.76 of model weight (Table 1). The most parsimonious model had an Akaike Information Criteria (AIC)^34^ weight of 0.32 and constant survival rates for males and females, probability of observation by tourists variable by time, and constant detection over the study period. Other parameters estimated by the Barker/RD model are found in Table A5.

**Table 1:** (A) Above: model results with the top 10 models from the Barker Robust Design Model.

Rank	Model	∆AICc	AICc weight	Model likelihood	K	−2log(L)
1	S(.) R(year) R′(sex) a′′(year) a′(.) p(.) pi	0.00	0.32	1.00	25	754.35
2	S(.) R(.) R′(sex*year) a′′(year) a′(.) p(.) pi	0.54	0.24	0.76	21	764.67
3	S(.) R(year) R′(sex) a′′(year) a′(.) p(.) pi TRANSIENCE	0.85	0.21	0.65	31	739.84
4	S(.) R(year) R'(sex) a′′(year) a′(.) p(.) pi	2.49	0.09	0.29	26	754.34
5	S(sex) R(sex) R'(sex) a′′(year) a′(.) p(.) pi	3.55	0.05	0.17	22	765.27
6	S(.) R(.) R′(year) a′′(year) a′(.) p(.) pi	4.15	0.04	0.13	22	765.87
7	S(.) R(sex*year) R′(sex) a′′(year) a′(.) p(.) pi	4.62	0.03	0.10	27	753.95
8	S(.) R(.) R'(sex) a′′(year) a′(.) p(.) pi	7.92	0.01	0.02	20	774.45
9	S(.) R(.) R′(sex*year) a′′(year) a′(.) p(.) pi TRANSIENCE	9.03	0.00	0.01	29	753.23
10	S(.) R(.) R′(.) a′′(year) a′(.) p(.) pi	9.23	0.00	0.01	19	778.13

Rank	Model	AICc	∆AICc	W	K	
1	SECR.σ	490.509	0.00	0.9	5	
2	SECR.sex	495.114	4.61	0.09	6	
3	SECR.0	499.597	9.09	0.01	4	
4	SECR.g0	501.519	11.01	0	5	

S, survival; R, observation by tourists; R′, dead but not recovered between primary periods; a′′, inside the study area given previously in the study area; a′, previously outside the study area; p, detection probability; pi, heterogeneity. (B) Model results from the spatially explicit capture-recapture density analysis. AICc, Akaike Information Criterion adjusted for small sample sizes; ΔAICc, difference in AICc values between each model and the model with the lowest AICc; W, AICc model weights; K, number of model parameters; g0, probability of capture at the home range centre; σ, spatial parameter related to home range size; SECR.g0, g0 varies between males and females; SECR.σ, σ varies between males and females; SECR.sex, both g0 and σ vary between males and females; SECR.0, null model.

Our second most parsimonious model, with 0.24 model weight, differed only by constant probability of observation by tourists. Our third model, with 0.21 model weight, demonstrated evidence of transience, whereby a jaguar was detected once in the study area and never again. Below we present estimates from the top model and model-averaged estimates for our parameters of interest. Model-averaged estimates for all parameters can be found in Table A5.

### Survival estimates

**Figure 2:**
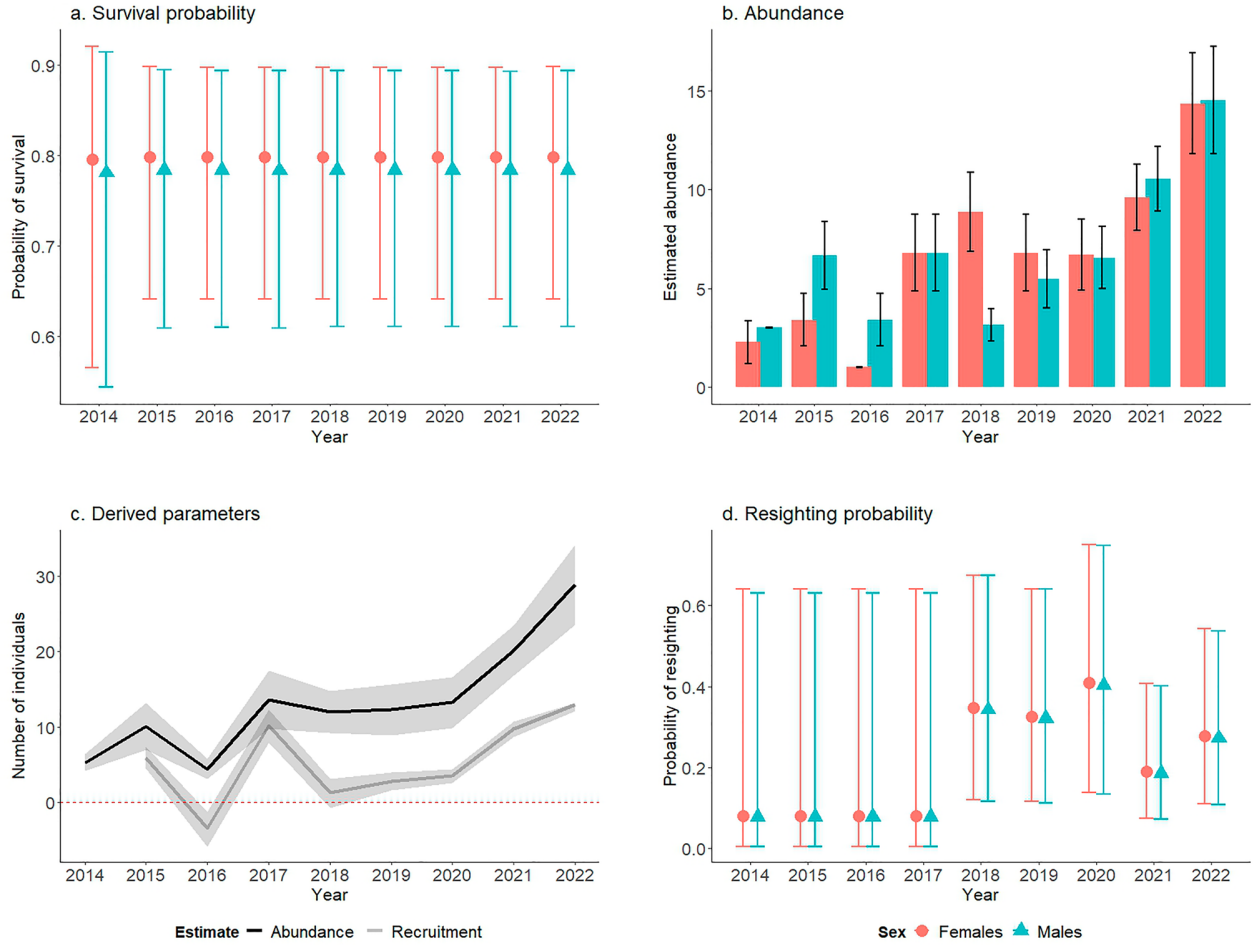
(**a**) Model weighted survival probability for male and female jaguars. (**b**) Model weighted derived estimates of male and female jaguar abundances. (**c**) Derived abundance estimates and Delta method results for recruitment. (**d**) Resighting probability by tourists. Error bars and shaded areas indicate 95% confidence intervals.

Our survival parameter was apparent survival because dead recoveries of individuals were not possible^28^. Apparent survival was constant throughout the study period (Fig. 2a), though the third most parsimonious model suggested transience, where an individual was detected once and did not return. Model-averaged estimates suggest a slight difference between males and females. The average survival rate was 0.783 (SE 0.075, 95% CI: 0.603–0.896) for males and 0.798 (SE 0.068 95% CI: 0.633–0.900) for females. Survival rates given transience were lower, 0.706 (SE 0.199, 95% CI: 0.318–0.934) for males and 0.720 (SE 0.200, 95% CI: 0.322–0.941) for females.

### Tourist observation estimates

The probability of observation by tourists varied over time (Fig. 2d). From 2014 to 2017, the probability of observation by tourists of both sexes was effectively zero. In 2018, the probability of observation by tourists increased to 0.348 (SE 0.157, 95% CI: 0.121–0.674) for females and 0.342 (SE 0.158, 95% CI: 0.116–0.674) for males. Observation by tourists peaked in 2020 at 0.409 (SE 0.181, 95% CI: 0.137–0.750) for females and 0.403 (SE 0.182, 95% CI: 0.133–0.748) for males. The observation probability dropped to less than half of that value in 2021, at 0.186 (SE 0.083, 95% CI: 0.072–0.400) for males and 0.190 (SE 0.084, 95% CI: 0.074–0.407) for females, before rebounding to 0.273 (SE 0.114, 95% CI: 0.108–0.537) for males and 0.279 (SE 0.115, 95% CI: 0.111–0.542) for females in 2022.

### Abundance estimates

Abundance increased more than five-fold over the nine-year period in Hato La Aurora (Fig. 2b and 2c). In 2014, there were estimated 3.00 males (SE 0.017, 95% CI: 2.97–3.03) and 2.63 females (SE 0.557, 95% CI: 0.12–3.36). The maximum estimated abundance in the nine-year period was in 2022, when males reached 14.54 individuals (SE 1.392, 95% CI: 11.81–17.27) and females reached 14.37 (SE 1.307, 95% CI: 11.81–16.93). The lowest recorded abundance was in 2016, when abundance was only four—1.00 female and 3.41 males (SE 0.0105, 95% CI: 0.98–1.02; SE 0.686, 95% CI: 0.2.06–4.74). Our derived abundance estimates differ from our raw numbers of jaguars, which suggests that we did not observe all jaguars that were present in Hato La Aurora (p* < 1) most years, with the exception of 2018. However, detection was high during the study period: 0.900 for mixture 1 of jaguars whose territory was mostly in the reserve and 0.412 for mixture 2 of jaguars whose territory partially overlapped in the top model.

### Recruitment and population growth from derived parameters

The Delta method for estimating derived parameters revealed an increasing population during the study; finite rate of change in abundance (λ) averaged 1.389 for males and 1.822 for females (Table A5). Notably, the female population had the highest growth between 2016 and 2017, when it rose to 6.801 because of the arrival of five new females. Male population growth was highest in the intervals of 2014–2015 (2.215) and 2016–2017 (2.000).

**Figure 3:**
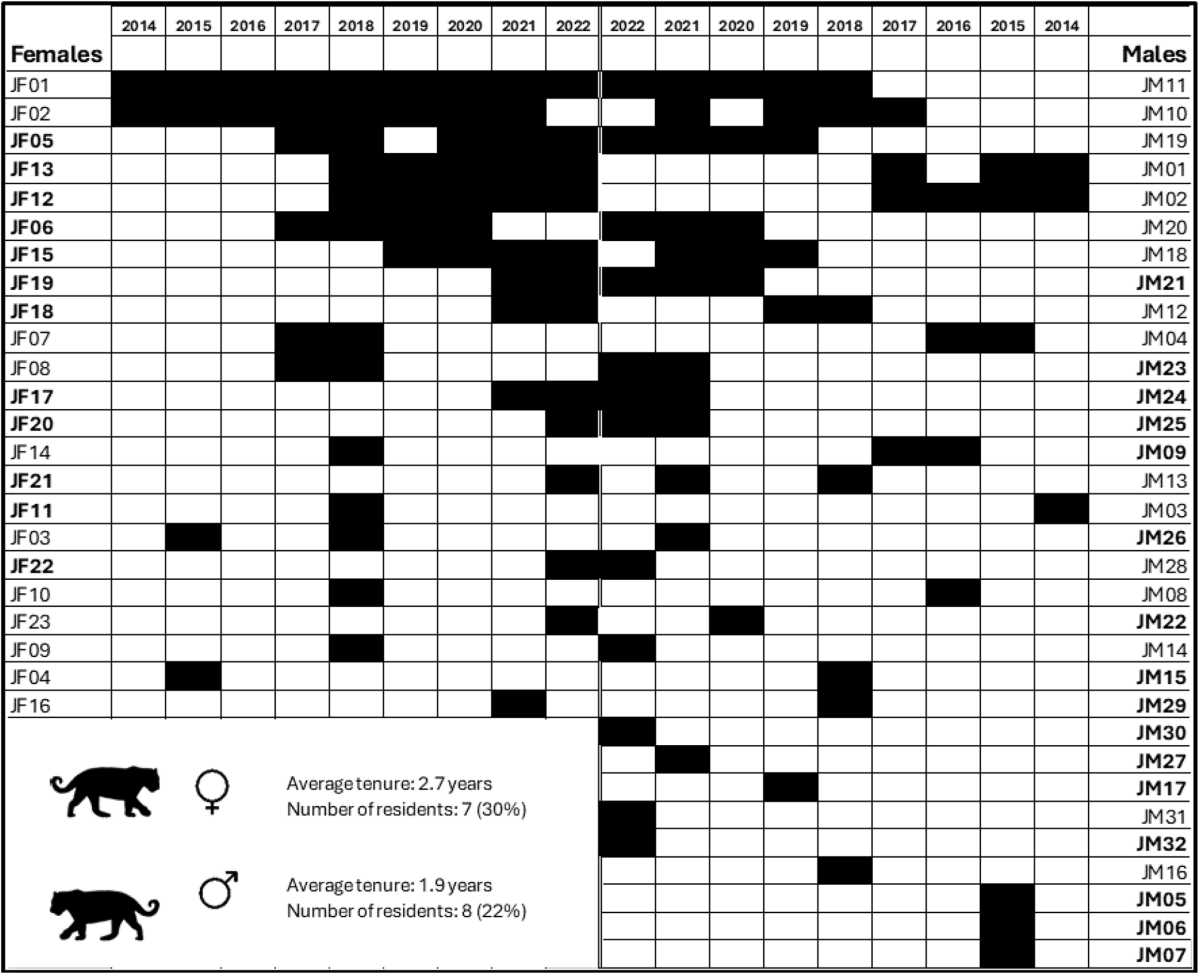
Life histories of jaguars detected in Hato La Aurora since 2014. Bolded names are jaguars that were born in Hato La Aurora. Jaguar icon credit: Gabriela Palomo-Muñoz.

Recruitment of both male and female jaguars was highest from 2021–2022, when it was 6.253 for males and 6.699 for females (Fig. 2c). The lowest recruitment for males (− 2.184) occurred between 2017 and 2018. The lowest recruitment for females (− 1.714) occurred between 2015 and 2016. Life histories of jaguars between 2014 and 2022 are shown in Fig. 3.

### SECR density

Trapping effort for spatially explicit capture-recapture (SECR) density in 2022 totaled 1985 camera trap nights. We recorded 17 individuals (99 capture events at 19 of 32 stations): nine females (49 capture events) and eight males (50 capture events). Seven females and seven males were captured at different stations. The best model was SECR.*σ* where *σ* varies between males and females (Table 1). SECR.*σ* produced a density estimate of 3.803 jaguars/100 km^2^ (SE 1.048; 95% CI: 2.238–6.464). The *g*_0_ parameter estimate was 0.018 (SE 0.003; 95% CI: 0.013–0.026). The *σ* estimate was 2.321 km (SE 0.274; 95% CI 1.842–2.924) for females and 5.091 (SE 1.031; 95% CI: 3.437–7.540) for males. Despite overlapping estimates confidence intervals (CIs), the estimated density in 2022 was approximately twice that from 2014 (1.88 ± 0.87 jaguars/100 km^2^; 95% CI: 0.79–4.48)^24^. Estimates for *σ* were also similar for females (females 2014: 2.327, SE 0.693 95% CI 1.315–4.119) and higher for males (males 2014: 1.426, SE 0.129 95% CI 1.195–1.701) than 2014 estimates indicating that the smaller grid size in 2022 did not bias density estimates.

## Discussion

Conserving large carnivores requires strategies beyond protected areas^2^. Working lands with adequate conservation measures can provide sufficient habitat to sustain resident jaguar populations that are comparable to those of protected areas^4^. Promoting coexistence can be facilitated by economic mechanisms like tourism that can ease the livelihood impacts of living with large carnivores. To inform such initiatives, there is a need for rigorous, long-term data collection to understand impacts to carnivore demography^35^. However, few long-term demographic studies exist for jaguars, hindering the evaluation of conservation efforts^30^. Where such studies exist, they tend to focus on protected areas^27–29^. Our longitudinal study of jaguars in Hato La Aurora, a working ranch and tourism destination in the Colombian Llanos, suggests that private lands with low-intensity cattle ranching and tourism can sustain an abundant jaguar population if combined with conservation actions such as hunting prohibitions, abundant prey^36^, conservation agreements with adjacent ranches^28^ and depredation reduction strategies in the form of electric fencing of calving pastures.

Our application of the Barker/RD, which integrated tourist photos into nine years of camera trapping data, provided much-needed demographic estimates for jaguars, including the first survival estimates for jaguars on working lands and in Colombia. The inclusion of tourist photos increased the precision of survival estimates^32^ and also allowed for quantification of jaguar sightings. Our estimates indicate that Hato La Aurora supported 28 ± 2.70 individual jaguars on the 15,000-hectare ranch in 2022, which is comparable to that of small protected areas within their range. In the federally protected Cockscomb Basin Wildlife Sanctuary in Belize, for example, estimated jaguar abundance peaked at 31 ± 4.77 individuals in 49,000 hectares^27^, an area more than three times the size of Hato La Aurora. Jaguars in Hato La Aurora had a high survival rate (0.78 ± 0.075), again similar to the highest estimated survival rate from Cockscomb Basin Wildlife Sanctuary in Belize (0.78 ± 0.05)^27^.

Our 2022 density estimate of 3.80 ± 1.08 jaguars/100 km^2^ in Hato La Aurora is consistent with recent density studies of jaguars from other working lands. In the Brazilian Pantanal, Devlin et al.^4^ estimated 4.08 ± 0.73 jaguars/100 km^2^ on multi-use (ranching, conservation, and tourism) landscapes. On a state-run cattle ranch in the Venezuelan Llanos with a long history of conservation, Jędrzejewski et al.^37^ estimated a density of 7.67 jaguars/100 km^2^. The ecological similarity of the Venezuelan Llanos with Hato La Aurora suggests that the Colombian Llanos could host a higher density of jaguars if threats are sufficiently reduced.

The increase in density estimates from 1.88 ± 0.87 in 2014 to 3.80 ± 1.08 jaguars/100 km^2^ in 2022, and the concomitant increase in jaguar abundance from five to 28 individuals, is encouraging for range-wide conservation efforts like the Jaguar Corridor Initiative, which seeks to maintain genetic connectivity between source populations throughout Central and South America^38^. This population increase was likely due to the high survival rate of jaguars and the 1.82 annual population growth (λ) of females, which is an important determinant of demography for long-lived species with low reproduction rates^39^. However, the specific causal mechanisms driving the population increase require further investigation. We speculate that the status of Hato La Aurora as a private reserve and the conservation actions (e.g., electric fences for calving pastures and conservation agreements) implemented by Panthera Colombia on smaller, nearby ranches enhanced habitat suitability for jaguars, reduced human hunting of prey, and decreased livestock depredations and therefore retaliatory killings. The distribution of land ownership in the Colombian Llanos, whereby large ranches are often surrounded by smaller parcels, may necessitate a dual approach to coexistence strategies. Actions like electric fencing to reduce depredations on larger ranches like Hato La Aurora may be cost-prohibitive because of the extension of land and size of cattle herds. Similarly, tourism on smaller ranches surrounding Hato La Aurora is challenging due to smaller plots of land and limited infrastructure, but electric fencing of pastures is more feasible. In addition, it is possible that jaguar populations are recovering throughout the region since hunting and pelt exports were outlawed following the inclusion of jaguars in the CITES Appendix I in the 1970s^31^. Tourism is the economic mechanism that allows the ranch owners to coexist with jaguars in Hato La Aurora, and therefore is of critical importance to jaguar persistence on the landscape.

Sustaining large carnivores on ranchlands requires minimizing livelihood impacts^7^. Tortato et al.^14^ found that in the Brazilian Pantanal income was over 50 times higher from tourism than the estimated cost of livestock depredation on cattle ranches. In Hato La Aurora, wildlife tourism is the economic vehicle that permits coexistence between livestock systems and jaguars, though the differential between tourism and livestock depredation is lower than that of the Brazilian Pantanal. Hato La Aurora loses on average 100 head of cattle, and a similar number of foals and pigs, to jaguars and pumas annually. Accurate data on income from tourism and the number of tourists visiting Hato La Aurora were unavailable, but ranch owners report that tourism income is crucial to offset the cost of living with carnivores. The allure of observing a jaguar in the wild raises the attractiveness of tourism in Hato La Aurora—further contributing to the viability of this coexistence strategy.

A challenge to large carnivore tourism, however, is reconciling their elusive nature with tourists’ desire for predictable, high-quality sightings^40^. Hunting prohibitions can make it easier to see species in tourist areas^41^, and savannahs are ideal observation sites because of visibility^42^. This appears to be the case in Hato La Aurora where our models showed higher probability of observation by tourists in recent years compared to 2014–2017. The probability peaked at 0.409 ± 0.182 in 2020 in the dry season months before the Covid-19 lockdown in March of 2020. The next year, in 2021, tourist sightings declined, which we attribute to lingering Covid-19 travel restrictions. We posit that, since jaguars in Hato La Aurora are not hunted or hazed, they may perceive a safer setting and be less likely to avoid human activity. Alternatively, a contributing factor to increased sightings may also be the knowledge of ranch owners and guides of daily patterns of jaguars. As tour guides came to understand where jaguars may be at peak times, they may have frequented those sites, leading to an increase in sighting probability.

Community science data collected by tourists, such as the photographs analyzed in our study, can have important and low-cost contributions to wildlife monitoring efforts^21^. Our application of the Barker/RD using tourist photos as auxiliary data can be applied to other monitoring programs of large, terrestrial mammals, especially to understand the probability of tourists observing them in the wild. When using tourist data, it would be useful to collect information on “sampling effort” of tourists by recording the number of people in each tour and the time spent observing wildlife and locations^21^. If using camera traps for robust design modeling, moving stations within and between survey periods may increase total detection probability of individual animals and reduce sex-specific and individual heterogeneity^27,43^.

Replicable and landscape-scale coexistence strategies are necessary for large carnivore conservation. In the case of jaguar tourism, understanding the causal mechanisms behind increased sightings is important to strengthen tourism in Hato La Aurora and beyond. Accompanying studies to assess tourism’s impact on attitudes and tolerance^44,45^ and local livelihoods^46^ are necessary for adaptive management of the industry and to optimize long-term benefits for jaguars. For jaguars in Colombia, prey depletion and the introduction of bovines into jaguar habitat are likely exacerbating conflict. Studies on jaguar diets, quantifying retaliatory killings, evaluating nonlethal strategies to reduce livestock depredation, and the human dimensions of living with jaguars are needed to understand barriers and enablers for human-jaguar coexistence at scale.

## Methods

### Study area

Colombia is the most diverse country by land area^47^, harboring 1941 species of birds^48^, 520 mammals^49^, and the third largest population of jaguars^23^. The department of Casanare, located in the country’s eastern *Llanos* (plains), is part of the transition zone between the tropical rainforest of the Amazon and the Eastern Andes. The landscape is dominated by seasonally flooded savannahs of the Orinoco basin, which form the largest wetland complex in the country. Casanare is a top destination for wildlife viewing due to open savannahs and abundant populations of mammals and birds. The area has been dominated by extensive cattle ranches since bovine introduction in the 1600s^50^. However, petroleum exploitation, oil palm plantations, and rice cultivation have increased in the last few decades^51^.

**Figure 4:**
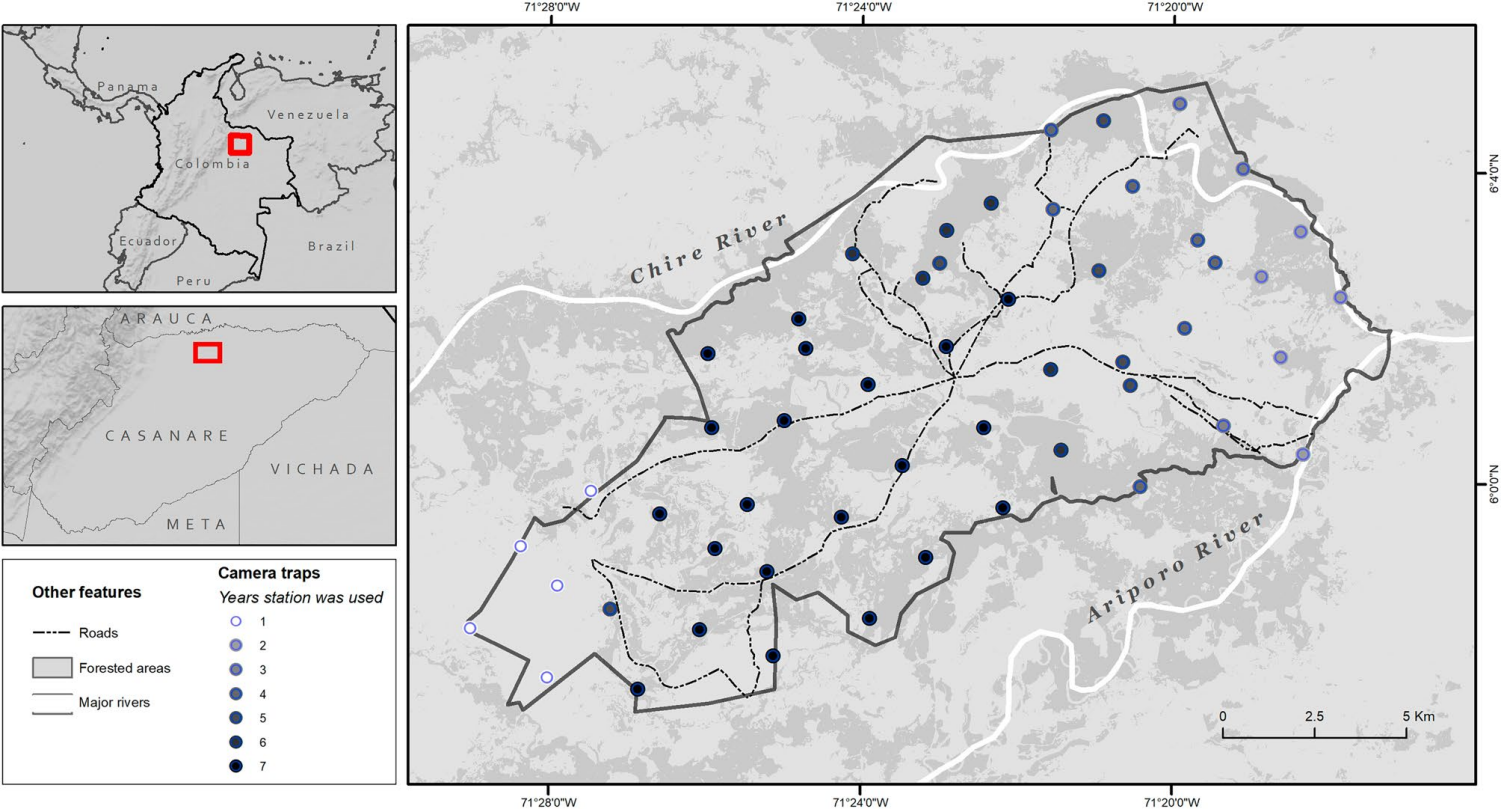
Location of Hato La Aurora, Colombia, and camera trap stations between 2014 and 2022. Sources: Natural Earth, U.S. Geological Survey, Panthera Colombia, Fundación Jaguar Colombia.

Hato La Aurora (5° 57′ 18.8″ N, 71° 29′ 0.1″ E to 6° 4′ 52.6″ N, 71° 17′ 51.4″ E) is a private reserve consisting of 15000 hectares of tropical savannahs and gallery forests in the department of Casanare (Fig. 4). The area receives 1000–3000 mm of rainfall each year, with marked dry (December–May) and wet (June–November) seasons^52^. The principal land use and economic activity is cattle ranching, with some introduced grasses for cattle forage. The surrounding area is comprised principally of extensively managed cattle ranches with riparian forest cover, though rice plantations are increasing in the region.

### Camera trapping

We installed a total of 296 camera-traps (models Panthera V3, V4, V5, V7, and Cuddeback 1279 and G-5048) between 2014 and 2022 at a distance of 1.5 ± 0.5 km for the study of medium and large vertebrates^53^ for a total of 16790 trap-nights (Table A1). Camera-trap grids used the same 24-h configuration and a quiet period of (30 s) between trigger events. No baits were used in any of the studies.

Camera trapping for density estimation (2014 and 2022 surveys) followed standardized recommendations^53^ and complied with the capture-recapture model assumptions: the population is closed, and all individuals have a possibility of being captured^54,55^. In April–May of 2014, we installed 52 double camera stations in a grid covering 152 km^2^ at an average distance of 1.6 ± 0.2 km (see Boron et al.^24^ for detailed survey information). In March–May of 2022, we placed 32 single camera stations (Table A6) in a grid covering 102 km^2^ (minimum convex polygon). We installed Cuddeback models 1279 and G-5048, and Panthera series 6 and 7 cameras at a height of 35–40 cm. Cameras remained active for 24 h per day. We used single stations because long-term monitoring of individuals provided photographic evidence of both sides of most jaguars, enabling individual identification when only one side was photographed. Like in 2014, the average distance between stations was 1.6 ± 0.2 km, which is consistent with recommendations for jaguar density studies^53^ and is appropriate when considering jaguar home ranges estimates, since it ensures all individuals can be photo-captured^56^. According to Colombian regulation, non-invasive camera trap studies do not require permits or approval from an Institutional Animal Care and Use Committee or equivalent.

### Auxiliary tourist photographs

Trained guides accompanied groups and ensured compliance with the regulations of the reserve. Jaguar viewing occurred in open vehicles and tour guides were required to maintain a minimum distance of 100 m, avoid any noises, and not leave the vehicle. We collected tourist photos of jaguars that were observed on an opportunistic basis during the study period. Tourists and guides reported sightings and delivered photos to co-author J. Barragan for identification. Jaguars were uniquely identified by rosettes and spot patterns^24^. Between 2014 and 2022, we collected 79 direct observations where individuals were identifiable. These sightings occurred primarily during the dry season (December–May) due to access issues in the rainy season and prey species like capybaras (*Hydrochoerus hydrochaeris*) being constricted to available surface water. However, a small number of sightings (*n* = *9*) occurred during the rainy season. Fifty-six additional sightings were discarded due to a lack of distinguishable photographic evidence.

### Barker/Robust design

We used a Barker/RD^32^ to estimate survival (*S*), detection probability by camera trap (*p*), availability to camera traps given previously inside the study area (*a*′′) and previously outside the study area (*a*′), probability of observation by tourists given alive (*R*) and probability of being dead but not recovered (*R*′), and abundance (*N*) of jaguars in Hato La Aurora. We extracted nine annual primary periods from camera trapping studies, eight of which occurred during the dry season and onset of the rainy season (between February and June) and one which occurred during the rainy season (July–October). We shortened camera trap studies to four-month secondary periods for closure^28^, as required by the model^32^. Camera trap records from outside of the closed period were removed from analysis. Details of the study periods can be found in Table A2.

Detection histories were compiled for each individual. Camera trap data were used for the secondary periods. Tourist photos, which could be collected at any time, were included as auxiliary resightings of jaguars at the end of each primary period in the detection history. The inclusion of tourist photos allowed for measurement of probability of viewing by tourists and increased the precision of survival estimates. We then constructed models using the Barker/RD model in Program Mark^57^, allowing for a mixture of detection probabilities^58^ and conditioning on at least one capture^59^ for a primary period. Dead recovery information was not available in our study. We therefore fixed parameters of the probability of dead recoveries (*r*) to 0 and fidelity (*F*) to 1 since all observations were within the study area. We also accounted for the variable time interval lengths between primary periods, rescaling all survival parameters to an annual basis. Hypotheses and parameter definitions can be found in Table A1.

We used a stepwise approach to model selection because of the large number of potential models associated with Barker/RD^28,60^. We began by fitting models of probability of detection (*p*) to test hypotheses of time and sex variation. We did not test for trap response (*c*) because of the non-invasive nature of remote cameras and the possibility of the model mistaking trap response for heterogeneity. We tested all models for heterogeneity with two mixtures because of jaguars’ territorial nature and the assumption that some individuals’ home ranges will only partially overlap with the study area, while others will be completely within it. We hypothesized that *p* increased by primary periods because researchers improved camera site identification and placement techniques, and that *p* varied by sex^61^. We tested for variation in *p* by primary period, secondary period, sex, and constant detection.

We modeled the availability parameters, which describe whether a jaguar was previously in the study area (*a*′′) or previously outside the study area (*a*′). We hypothesized that the probability of being previously outside the study area (*a*′) and inside the study area (*a*′′) would be Markovian and be the result of sex and time interactions, whereby males were more likely to emigrate and immigrate than females because of dispersal and territoriality^62^. We compared models with no movement, random movement, and Markovian movement.

The resighting parameter in this version of the Barker/RD calculates the probability that each jaguar is observed directly by a tourist that year. We fit models to the probability of resighting (*R*), testing sex, time, sex and time interaction, linear trend, and constant models. Given the increase in jaguar sightings reported by tourists in the last 5 years, we hypothesized that resightings increased with time since 2017 and varied by sex, given that males are more likely to use higher risk areas^63^. Resighting effort is an unaccounted covariate in our study, since tour operators did not record the number of tours, hours of effort, or number of viewers.

Finally, we tested models for survival based on our hypothesis that survival would increase with time. We evaluated models with time variation, time and sex interaction, sex, and constant survival probabilities. Previous studies have applied prey density covariates to the survival parameter of robust design frameworks^64^. However, prey data were not available for all years of the study, thus could not be applied to survival. We selected the most parsimonious models using AIC for each hypothesized model^34^. We used model averaging for the estimated parameters above and the derived parameter of abundance (*N*).

### Derived parameters from the Delta method

We used the Delta method to calculate population changes over time^27,33^ and identify the source of abundance increases. We used derived parameter estimates of *N* to calculate the finite rate of change in abundance between sampling periods (λ), as *N*_*t*+*1*_/*N*_*t*_. We calculated the number of new recruits in the population in Hato La Aurora as *N*_*t*+*1*_−*N*_*t*_* φ* where *φ* is survival at time *t* + *1*^33^.

### Spatially explicit capture recapture

We ran density analysis with 2022 data to complement robust design estimates and compare with a density survey in Hato La Aurora in 2014^24^. We conducted density analysis fitting SECR models in a maximum likelihood framework^65,66^ in the R package “secr”^67^. SECR models identify individuals home range centers based on their spatial locations, then estimate density of these centers across an area that includes the camera grid^68,69^. In addition to the standard capture-recapture assumptions, SECR models assume circular and constant home ranges during the survey, randomly distributed home range centers, and that the encounter rate of an individual with a trap decreases with increasing distance from the home range center following a predefined function^68^. We used the half-normal detection function where the probability of capture (*p*) of an individual (*i*) at a trap (j) decreases with distance (*d*) from the activity center as: *P*_*ij*_ = *g*_*0*_* exp(− d*_*ij*_^*2*^*/2σ*^*2*^*)*. The parameter *g*_0_ is the probability of capture when the trap is located exactly at the center of the home range, and sigma (*σ*) is parameter of the spatial scale over which detection declines away from the home range center^68^. As appropriate for camera trap data, we deployed the binomial encounter model (or Bernoulli model), enabling individuals to be captured at different camera stations during one sampling occasion (i.e., 24-h period) but only once at each station^70,71^. Like other felids, jaguars have different behavior and home ranges between sexes, hence we allowed both parameters *g*_0_ and *σ* to vary with sex of the individuals^53,61^ and compared four models using the AIC: the null model (SECR.0), a model where *g*_0_ varies between males and females (SECR.*g*_0_), a model where *σ* varies between males and females (SECR.*σ*), and a model where both g0 and σ vary between sexes (SECR.sex).

## Supplementary Information

Supplementary Tables.

## Data Availability

The datasets for the SECR analysis are available in the appendices and all other data are available from the corresponding author on reasonable request.
