# Supplementary material for: Tourism-supported working lands sustain a growing jaguar population in the Colombian Llanos
Source: Sci Rep. 2023 Jun 27;13:10408. doi: 10.1038/s41598-023-36935-2 (PMC10300083; doi:10.1038/s41598-023-36935-2)

**Table A1. Hypotheses and parameters for the Barker Robust Design Model for jaguars in Hato La Aurora from 2014-2022.**

| **Parameter** | **Parameter description** | **Variable in models** | **Model description** |
| --- | --- | --- | --- |
| *S** | Probability that individual *i* in primary sampling period *t* survives to period *t + 1.* | S(.) | Constant apparent survival |
|  |  | S(sex) | Apparent survival based on sex |
|  |  | S(sex*time) | Apparent survival depends on sex and time interactions |
|  |  | S(time) | Survival depends on time |
| *p** | Probability that an individual *i* is detected in primary period *t*, given that it is alive, in the population, and available for detection. | p(.) | Constant detection probability |
|  |  | p (year) | Detection probability changes with session |
|  |  | p (sex) | Sex specific detection |
|  |  | p (year*sex) | Time and sex differences in detection |
| *π¹* | Heterogeneity in recapture probabilities based on individual characteristics | Mixture 1 | Group of individuals whose home range mostly occurs outside of the reserve |
|  |  | Mixture 2 | Group of individuals whose home range mostly falls within the core of the reserve |
| *R** | Probability that an individual *i* is detected alive and reported between primary periods *t* and *t + 1*, given it survives to period *t + 1*. | R(.) | Constant resighting probability |
|  |  | R(time) | Resighting probability changes over time |
|  |  | R(sex) | Resighting probability is different based on sex |
|  |  | R(sex*time) | Resighting probability depends on sex and time interactions |
|  |  | R(T) | Resighting probability fitted with a linear trend |
| *R’** | Probability that individual *i* is detected alive and reported between primary periods *t* and *t + 1*, given that it dies in that interval but is not recovered and reported. | R'(.) | Constant probability of being seen though dead and not reported |
|  |  | R’(time) | Time dependent probability |
|  |  | R’(sex) | Sex dependent probability |
|  |  | R’(sex*time) | Sex and time interaction for the probability |
| *a'** | Probability individual *i* is available for detection in primary period *t + 1*, given it was unavailable in period t, and survived and remained faithful to the population from *t* to *t + 1*. | a'(.) | Random movement |
|  |  | a’(time) | Markovian movement |
|  |  | a’(sex*t) | Markovian movement based on sex and time interactions |
|  |  | a’(sex) | Markovian movement based on sex |
| *a”** | Probability individual *i* is available for detection in primary period *t + 1*, given it was available in period *t*, and survived and remained faithful to the population from period *t* to *t + 1*. | a"(=1) | No movement |
|  |  | a"(time) | Time varying movement (Markovian) |
|  |  | a"(sex) | Movement varying by sex |
|  |  | a"(sex*time) | Movement varying by sex and time interactions |
| N* | The size of the subset of the population that is available in the study area during primary period *t*. | Derived parameter | |
|  |  |  | |

¹ All models included heterogeneity in detection probably because of the selected data type (Huggins with heterogeneity and *p*).

* Parameter descriptions from Kendall et al. 2013

**Table A2. Camera trapping effort during the study period.**

**Table A3. SCR capture history in 2022.**

**Session is the survey in 2022, Animal_ID = Individual jaguar, SO = survey occasion, LOC_ID = camera location ID.**

| SESSION | ANIMAL_ID | SO | LOC_ID | SEX |
| --- | --- | --- | --- | --- |
| 1 | 1 | 73 | 2 | F |
| 1 | 1 | 84 | 5 | F |
| 1 | 2 | 3 | 16 | F |
| 1 | 2 | 8 | 16 | F |
| 1 | 2 | 25 | 28 | F |
| 1 | 2 | 27 | 28 | F |
| 1 | 2 | 28 | 28 | F |
| 1 | 2 | 29 | 28 | F |
| 1 | 2 | 71 | 28 | F |
| 1 | 3 | 18 | 33 | F |
| 1 | 3 | 25 | 28 | F |
| 1 | 3 | 30 | 5 | F |
| 1 | 3 | 37 | 5 | F |
| 1 | 3 | 52 | 2 | F |
| 1 | 3 | 73 | 2 | F |
| 1 | 3 | 81 | 28 | F |
| 1 | 3 | 84 | 5 | F |
| 1 | 4 | 18 | 33 | M |
| 1 | 4 | 4 | 7 | M |
| 1 | 4 | 26 | 7 | M |
| 1 | 4 | 29 | 16 | M |
| 1 | 4 | 57 | 16 | M |
| 1 | 5 | 2 | 28 | M |
| 1 | 5 | 37 | 28 | M |
| 1 | 5 | 44 | 28 | M |
| 1 | 5 | 46 | 21 | M |
| 1 | 5 | 48 | 38 | M |
| 1 | 5 | 51 | 16 | M |
| 1 | 5 | 57 | 28 | M |
| 1 | 5 | 61 | 28 | M |
| 1 | 5 | 62 | 23 | M |
| 1 | 5 | 62 | 16 | M |
| 1 | 5 | 65 | 28 | M |
| 1 | 5 | 67 | 23 | M |
| 1 | 5 | 67 | 24 | M |
| 1 | 5 | 74 | 23 | M |
| 1 | 5 | 86 | 16 | M |
| 1 | 5 | 87 | 23 | M |
| 1 | 5 | 90 | 16 | M |
| 1 | 5 | 70 | 2 | M |
| 1 | 5 | 72 | 7 | F |
| 1 | 6 | 44 | 17 | M |
| 1 | 6 | 51 | 12 | M |
| 1 | 6 | 81 | 24 | M |
| 1 | 7 | 5 | 4 | M |
| 1 | 7 | 53 | 12 | M |
| 1 | 7 | 54 | 8 | M |
| 1 | 7 | 74 | 8 | M |
| 1 | 7 | 86 | 3 | M |
| 1 | 8 | 7 | 23 | F |
| 1 | 8 | 1 | 33 | F |
| 1 | 8 | 25 | 28 | F |
| 1 | 8 | 85 | 2 | F |
| 1 | 8 | 86 | 2 | F |
| 1 | 9 | 1 | 28 | F |
| 1 | 9 | 3 | 28 | F |
| 1 | 9 | 84 | 28 | F |
| 1 | 9 | 89 | 2 | F |
| 1 | 10 | 1 | 16 | M |
| 1 | 10 | 1 | 28 | M |
| 1 | 10 | 27 | 28 | M |
| 1 | 10 | 28 | 28 | M |
| 1 | 10 | 29 | 28 | M |
| 1 | 10 | 66 | 14 | M |
| 1 | 10 | 81 | 14 | M |
| 1 | 10 | 89 | 7 | M |
| 1 | 11 | 3 | 7 | F |
| 1 | 11 | 49 | 3 | M |
| 1 | 12 | 2 | 33 | F |
| 1 | 12 | 29 | 16 | F |
| 1 | 12 | 57 | 16 | F |
| 1 | 13 | 2 | 25 | F |
| 1 | 13 | 5 | 25 | F |
| 1 | 13 | 63 | 26 | F |
| 1 | 13 | 72 | 36 | F |
| 1 | 13 | 81 | 16 | F |
| 1 | 14 | 20 | 3 | M |
| 1 | 14 | 25 | 7 | M |
| 1 | 14 | 29 | 17 | M |
| 1 | 14 | 38 | 17 | M |
| 1 | 14 | 39 | 17 | M |
| 1 | 14 | 42 | 17 | M |
| 1 | 15 | 73 | 2 | M |
| 1 | 15 | 84 | 5 | M |
| 1 | 16 | 1 | 33 | F |
| 1 | 16 | 4 | 7 | F |
| 1 | 16 | 7 | 7 | F |
| 1 | 16 | 8 | 7 | F |
| 1 | 16 | 13 | 24 | F |
| 1 | 16 | 29 | 16 | F |
| 1 | 16 | 45 | 7 | F |
| 1 | 16 | 46 | 16 | F |
| 1 | 16 | 54 | 7 | F |
| 1 | 16 | 54 | 24 | F |
| 1 | 16 | 57 | 16 | F |
| 1 | 16 | 65 | 16 | F |
| 1 | 16 | 65 | 24 | F |
| 1 | 17 | 6 | 4 | M |
| 1 | 17 | 56 | 3 | M |

**Table A4. SCR station information for jaguars in 2022.**

**Loc= Camera trap location. X and Y indicate UTM 19N coordinates of stations. 1 indicates camera was operational, - indicates it was non-operational.**

**Table A5. Model averaged estimates from the Barker Robust Design Model.**

**A.**

B.

**C.**

**D.**

**E.**

**F.**

**Table A6. 2022 SCR study map**


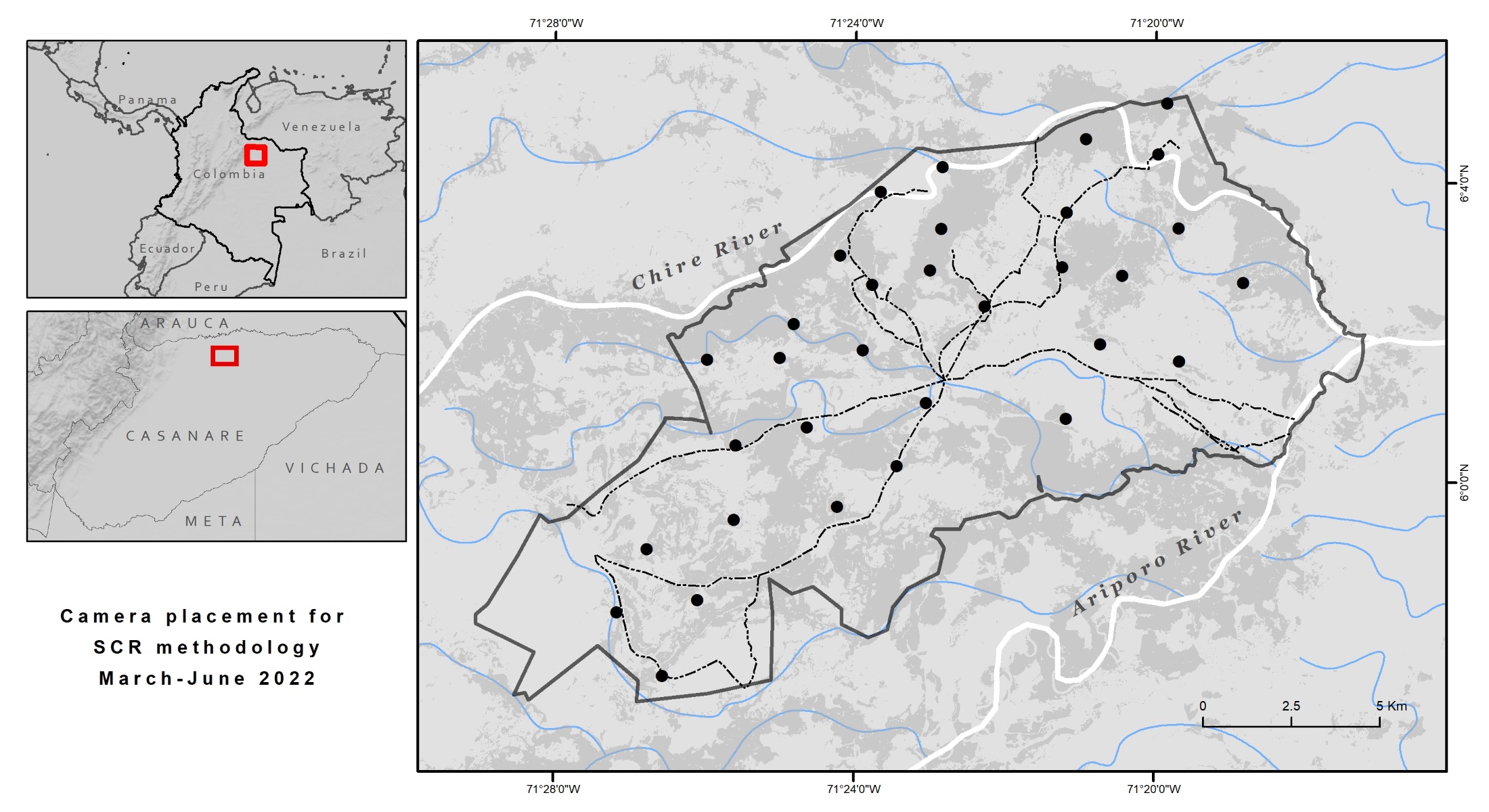

Supplement: Supplementary file 1 — Supplementary Tables. [file 41598_2023_36935_MOESM1_ESM.docx]
